# Acellular dermis (SureDerm^®^) use for managing pulsatile tinnitus: a long-term follow-up of a novel surgical technique

**DOI:** 10.3205/iprs000156

**Published:** 2021-06-09

**Authors:** Joshua Agilinko, Sara Katharine Drever, Winston Kin Wai Low, Muhammad Shakeel, Akhtar Hussain

**Affiliations:** 1 Department of Otolaryngology, Head and Neck Surgery, Aberdeen Royal Infirmary, Aberdeen, United Kingdom

## Abstract

**Introduction:** Pulsatile tinnitus (PT) can be very distressing for the patient. An identifiable abnormality is rarely detected. Dural AV malformation is responsible for arterial PT. Venous PT has rarely been attributed to an obvious abnormality on venogram. Dehiscent high jugular bulb or sigmoid sinus have been thought to be potential cause for venous PT. Ligation of internal jugular vein (IJV) has been advocated as a definitive surgical treatment. To our knowledge the use of acellular dermal matrix for treatment of venous PT has not been reported previously.

**Objectives:** To share our experience of a successful treatment of PT using acellular dermis.

**Methodology:** Case report and literature review.

**Case description:** A 23-year-old Caucasian female presented with right-sided PT of 9 months duration. All clinical and audiological investigations were normal. MRI brain and internal auditory canals was normal but the CT scan showed a high right jugular bulb. It also showed dehiscence of the right sigmoid plate with herniation of sigmoid sinus into the mastoid. She underwent transmastoid correction of dehiscent sigmoid sinus and jugular bulb. Acellular dermis was used for extra luminal packing of mastoid cavity and hypotympanum. The patient made a good post-operative recovery and reported resolution of tinnitus on recovering from anaesthesia. The patient was discharged home the following day. There were no sequelae from surgery. The patient has remained symptom-free 11 years following her treatment.

**Conclusion:** The surgical goal of dehiscent sigmoid sinus correction can be accomplished with acellular dermis packing. Traditionally ligation of the IJV or rigid correction of herniated sinus has been recommended; however, we have demonstrated that a relatively thick pliable acellular dermis is more than adequate to correct herniation of the sigmoid venous sinus.

## Zusammenfassung

**Einleitung:** Pulsierender Tinnitus (PT) kann für den Patienten sehr belastend sein. Selten wird eine identifizierbare Anomalie gefunden. Eine durale arteriovenöse Fehlbildung ist für den arteriellen PT verantwortlich. Der venöse PT wurde selten auf eine offensichtliche Anomalie im Venogramm zurückgeführt. Eine Dehiszenz des Bulbus jugularis internus oder des Sinus sigmoideus wurde als mögliche Ursache für eine venöse PT angesehen. Die Ligatur der Vena jugularis interna wurde als maßgebliche chirurgische Behandlung empfohlen. Unseres Wissens nach wurde bisher noch nicht über die Verwendung einer azellulären dermalen Matrix zur Behandlung der venösen PT berichtet.

**Ziele:** Wir möchten unsere Erfahrungen mit einer erfolgreichen Behandlung von PT mit azellulärer Dermis teilen.

**Methodik:** Fallbericht und Literaturübersicht.

**Fallbeschreibung:** Eine 23-jährige kaukasische Frau stellte sich mit einem rechtsseitigen PT von 9 Monaten Dauer vor. Alle klinischen und audiologischen Untersuchungen waren normal. Das MRT des Gehirns und der inneren Gehörgänge war normal, aber der CT-Scan zeigte einen hohen rechten Jugularbulbus. Außerdem zeigte sich eine Dehiszenz der rechten Sigmaschale mit Hernie des Sinus sigmoideus in das Mastoid. Sie unterzog sich einer transmastoidalen Korrektur des dehiszenten Sinus sigmoideus und des Bulbus jugularis. Acelluläre Dermis wurde zur extraluminalen Auffüllung der Mastoidhöhle und des Hypotympanums verwendet. Der Patient erholte sich postoperativ gut und berichtete über ein Verschwinden des Tinnitus nach dem Aufwachen aus der Anästhesie. Die Patientin wurde am nächsten Tag nach Hause entlassen. Es gab keine Folgeerscheinungen der Operation. Die Patientin ist 11 Jahre nach der Behandlung symptomfrei geblieben.

**Schlussfolgerung:** Das chirurgische Ziel der Dehiszenzkorrektur des Sinus sigmoideus kann mit azellulärer Dermispackung erreicht werden. Traditionell wurde die Ligatur der IJV oder eine starre Korrektur des Sinus herni empfohlen. Wir haben jedoch gezeigt, dass eine relativ dicke, biegsame azelluläre Dermis mehr als ausreichend ist, um die Hernie des Sinus sigmoideus zu korrigieren.

## Introduction

Pulsatile tinnitus (PT) can be very distressing for the patient. An identifiable abnormality is rarely detected. Vascular disorders including arteriovenous fistula, internal carotid artery aneurysm and dissection account for half of arterial PT [1]. Dehiscent high jugular bulb or sigmoid sinus on CT scan have been thought to be potential causes of venous PT [2]. Angiography (arterial and venous) guides diagnosis and treatment.

Current treatment of PT incorporates reassurance of the patient, rehabilitation and cognitive behavioural therapies with variable success rates [3]. Ligation of internal jugular vein (IJV) and radical surgeries such as mastoidectomy have been advocated as definitive surgical treatment [4]. However these are associated with significant morbidity. 

Acellular dermal matrices (ADMs) are natural scaffolding with the potential to facilitate tissue regeneration. Since their introduction in resurfacing of burn injuries in the mid 1990s [5] their use now spans most surgical subspecialties, such as cleft palate repair and breast reconstruction [6].

ADMs undergo stringent technological processing to allow transplantation without rejection. Their role in otology has only been experimental in a guinea pig model [7]. To our knowledge the use of acellular dermis for treatment of venous PT has not been reported previously. The authors describe a surgical method based on human dermal allograft (SureDerm™, Hans Biomed Corp. Korea) for the repair of dehiscent jugular bulb and sigmoid sinus.

## Case description

A 23-year-old Caucasian female presented with right-sided pulsatile tinnitus of 9 months duration. She described hearing a whooshing noise. She did not have any hearing loss or dizziness. The patient struggled to focus while studying because of the intrusive tinnitus. 

**Figure 1:**
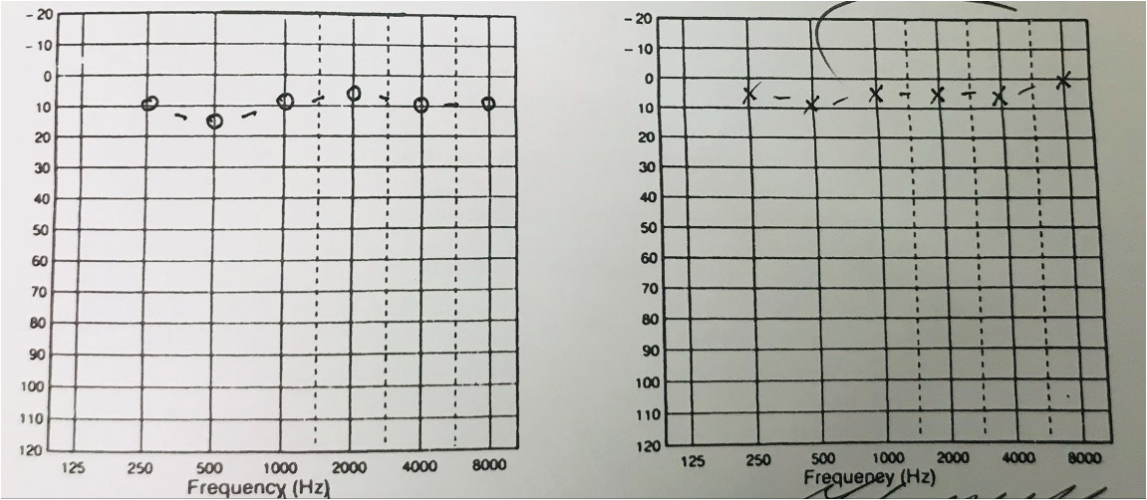
Preoperative pure tone audiogram revealing normal hearing thresholds in both ears

**Figure 2:**
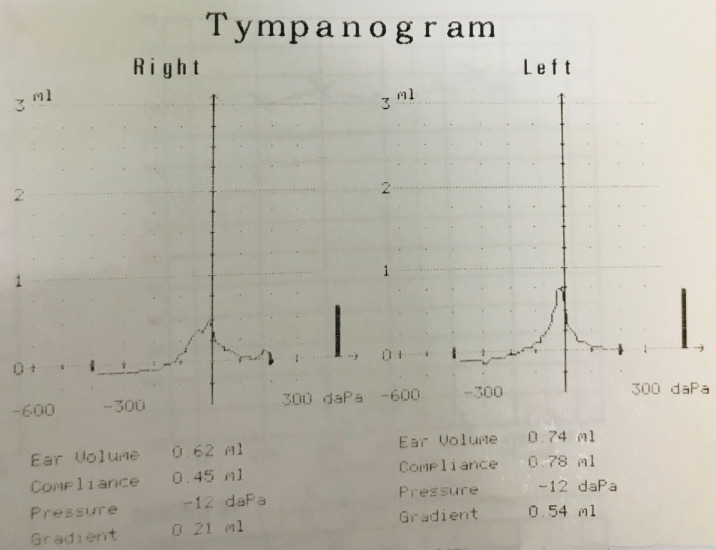
The preoperative tympanogram shows type A curve bilaterally.

The patient was initially seen in an otology clinic where she did not have any otological, rhinological or neurological symptoms. Otoscopy revealed normal tympanic membranes; cranial nerves were intact; cerebellum function was intact and the balance tests were negative. Full examination of the nose, mouth, pharynx and larynx was normal. Fundoscopy and auscultation of her head and neck were normal. Her blood pressure was 120/64 mmHg (millimetres of mercury). She had normal hearing on pure audiogram (Figure 1 (Fig. 1)) and normal ventilation of her middle ears on tympanogram (Figure 2 (Fig. 2)). 

**Figure 3:**
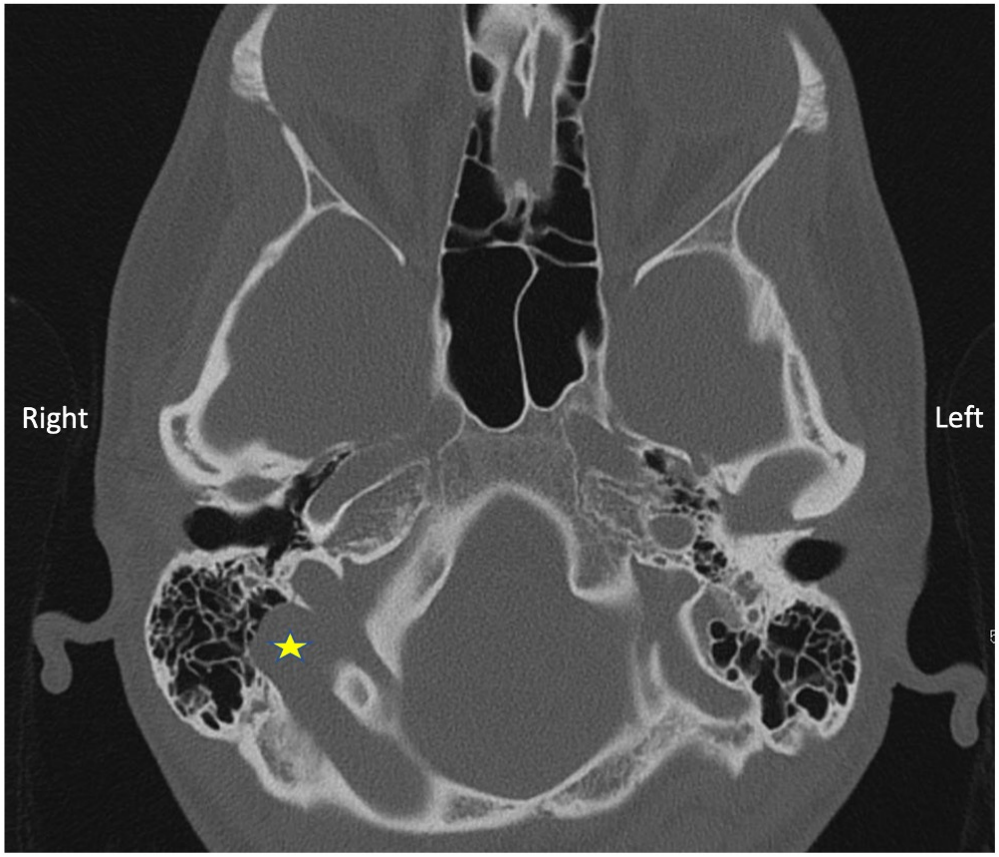
CT scan showing the right high jugular bulb, dehiscent sigmoid plate with sigmoid sinus herniation into the mastoid (yellow star)

All the routine blood tests including thyroid function tests were normal. MRI brain and internal auditory canals was normal. The CT scan of the petrous temporal bone showed a large high lying right-sided jugular bulb. It also showed dehiscence of right sigmoid plate with herniation of the lateral aspect of the transverse and sigmoid venous sinuses into the mastoid complex (Figure 3 (Fig. 3)). 

She was seen in a Tinnitus clinic and thorough counselling was provided by the senior audiologist. She tried environment sound therapy along with other conservative measures but continued to struggle with her intrusive right sided pulsatile tinnitus. 

The patient was seen by the skull base surgeon. Clinically, there was no dehiscence of the jugular bulb and the hypotympanum was clear. The patient described hearing a whooshing noise and it was accepted as a venous hum secondary to dehiscence of the sigmoid sinus. The presence of the high lying jugular bulb on radiology added complexity to the possible aetiology of her tinnitus. It was discussed that even correction of the sigmoid sinus dehiscence may not lead to symptom resolution as high lying jugular bulb is associated with tinnitus as well. The patient accepted this possibility and it was agreed that if her symptoms fail to settle down after dehiscent sigmoid correction then she might need ligation of the right internal jugular vein at a later stage. 

**Figure 4:**
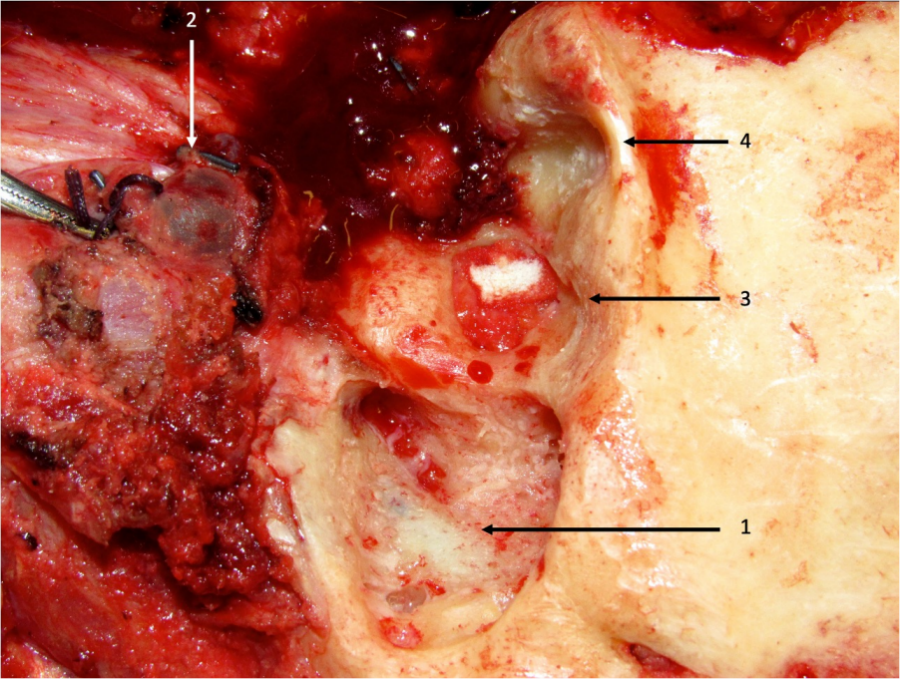
An example of intraoperative view of left cortical mastoidectomy showing sigmoid sinus (1). The patient is positioned supine with head turned to right. The patient is undergoing left lateral temporal bone resection, total parotidectomy, mandible resection, left neck dissection and reconstruction for malignancy. The left internal jugular vein has been ligated in the upper neck (2). The external auditory canal has been excised along with removal of the tympanic membrane and middle ear contents followed by obliteration of the middle ear with fat and gel foam (3). The glenoid or mandibular fossa can be seen clearly (4).

Surgical intervention: the patient was positioned supine with head elevation and turned to left. The skin was cleaned with betadine and standard drapes were applied. Postauricular incision was made exposing the mastoid bone. Cortical mastoidectomy was carried out to expose the sigmoid sinus, lateral semicircular canal and the jugular bulb. An example of intra-operative appearance of the cortical mastoidectomy and sigmoid sinus is shown in Figure 4 (Fig. 4). 

Intraoperatively, dehiscence was noted between the walls of the jugular bulb and floor of the middle ear. Bleeding encountered when separating the jugular bulb from the adjacent bones. The bleeding was controlled by Fibrillar and Surgicel packing. This was further supported by extraluminal packing with acellular dermal graft SureDerm. The jugular bulb was separated from the middle ear floor and packing with SureDerm continued between the sigmoid sinus and bony wall anteriorly. Multiple layers of SureDerm were used for extra luminal packing of mastoid cavity and hypotympanum along with Surgicel. Little muscle harvested from incision margin was placed on top of packing. The incision was closed with 3/0 monocryl and skin staples. Bactroban ointment was applied to the incision edges followed by gauze and crepe bandage. The microscope was used to examine the ear canal and tympanic membrane confirming absence of any haemotympanum. 

The patient made a good postoperative recovery and reported resolution of tinnitus on recovering from anaesthesia. The patient was discharged home the following day and head bandage was removed. 

Two days after the operation the patient noticed some bleeding from her nose and throat. On examination no active bleeding was seen from the ear and the nasal endoscopy did not show any bleeding from her nose or eustachian tube orifice. There was evidence of haemotympanum on microscopic examination of her right ear. The patient reported impaired hearing in her right ear. She was advised to avoid any physical exertion for the next few days. 

**Figure 5:**
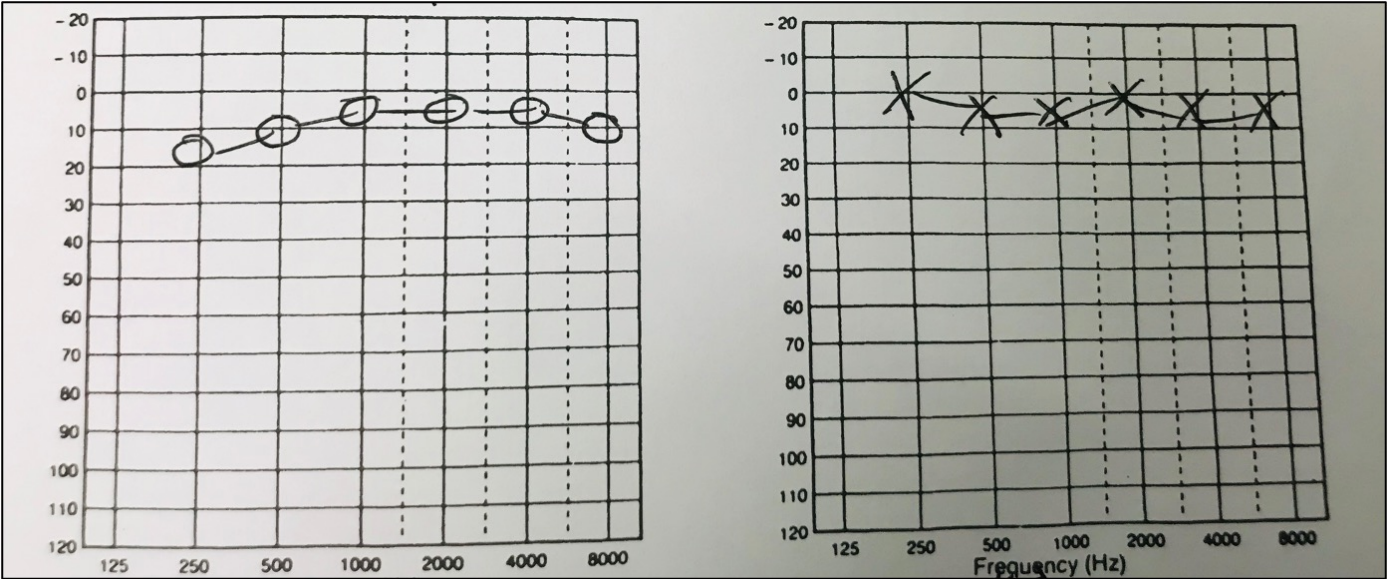
Postoperative pure-tone audiogram confirming normal hearing levels in both ears

At three-month follow-up the haemotympanum had resolved and her hearing had returned back to normal (Figure 5 (Fig. 5)). The patient has remained tinnitus free 11 years following her surgery. 

## Discussion

Tinnitus describes distressing otological symptoms associated with the perception of sound without corresponding acoustic processing in the cochlea [8]. Pulsatile tinnitus accounts for less than 10% of tinnitus [9]. Two possible causes of PT have been postulated:

1. turbulence theory which suggests that acceleration in blood flow results in local turbulence;
2. abnormal sound conduction (bone or air) leads to loss of the masking effect of external sounds [10].

Waldvogel and colleagues carried out a study of 84 patients over a 10-year period looking at causes of PT. Arteriovenous fistula, aneurysm and dissection of the internal carotid artery, fibromuscular dysplasia were found in 36 (42%) of patients [1]. In contrast, high jugular bulb with or without a diverticulum, stenosis of sigmoid sinus, on CT scan have been reported in venous PT [2]. It is believed that these anomalies are associated with turbulent blood flow near the middle ear, leading to subjective venous-type PT [11].

Non-surgical treatment of PT incorporates reassurance of the patient, rehabilitation and cognitive behavioural therapies with variable success rates [3]. 

The surgical goals in venous PT are to reduce the turbulent flow through the dehisced jugular bulb and sigmoid sinus. Surgical treatment currently involves ligation of the high jugular vein and mastoidectomy. However, postoperative complications have been reported in some cases including intracranial hypertension and sinus thrombosis [12].

Acellular dermal matrices (ADMs) were initially described for use in the resurfacing of burn injuries in 1995 [5]. Over the past decade, growth in the ADM market has been explosive. This has been due to increased surgeon and patient awareness and favourable clinical outcomes. Their use now spans many surgical subspecialties, including cleft palate repair and breast reconstruction [6]. 

ADMs are best regarded as natural acellular scaffolds which facilitate tissue healing. They are composed of collagen and extracellular matrix components. The ideal ADMs undergo several technological processing steps involving decellularisation, disinfection, dehydration and sterilisation [13]. These steps allow easy transplantation without rejection and infection. 

In otological surgeries, ADMs have only been used in a guinea pig model [7]. To the best of our knowledge, our case is the first time ADMs have been used in PT. 

Our patient had dehiscence of her right sigmoid plate with herniation of sigmoid sinus into the mastoid. In addition, she also had a high jugular bulb with dehiscence. She therefore underwent transmastoid correction of the dehisced sigmoid sinus and jugular bulb using acellular dermis for extra luminal packing of mastoid cavity and hypotympanum. The patient made a good post-operative recovery and reported disappearance of her tinnitus on recovering from her anaesthetic. 

The senior author has used this technique in another 4 patients with success and would recommend others to consider this option when dealing with such scenarios.

## Conclusion

The surgical goal of correcting the dehiscent sigmoid sinus can be accomplished with extraluminal packing using acellular dermis. Traditionally ligation of the IJV or rigid correction of herniated sinus has been recommended; however, we have demonstrated that a relatively thick pliable acellular dermis is more than adequate to correct herniation of the sigmoid venous sinus. We have no hesitation to recommend this treatment option to colleagues.

## Notes

### Competing interests

The authors declare that they have no competing interests.
